# IgE-Reactivity Pattern of Tomato Seed and Peel Nonspecific Lipid-Transfer Proteins after *in Vitro* Gastrointestinal Digestion

**DOI:** 10.1021/acs.jafc.0c06949

**Published:** 2021-03-15

**Authors:** Laura Martín-Pedraza, Cristobalina Mayorga, Francisca Gomez, Cristina Bueno-Díaz, Natalia Blanca-Lopez, Miguel González, Mónica Martínez-Blanco, Javier Cuesta-Herranz, Elena Molina, Mayte Villalba, Sara Benedé

**Affiliations:** † Department of Biochemistry and Molecular Biology, Universidad Complutense de Madrid, 28040 Madrid, Spain; ‡ Allergy Research Laboratory, IBIMA, Regional University Hospital of Málaga, UMA, 29009 Málaga, Spain; § Servicio de Alergia, Hospital Universitario Infanta Leonor, 28031 Madrid, Spain; ∥ Fundación IIS-Fundación Jiménez Díaz, 28040 Madrid, Spain; ⊥ Instituto de Investigación en Ciencias de la Alimentación (CIAL, CSIC-UAM), Nicolás Cabrera 9, 28049 Madrid, Spain

**Keywords:** tomato allergy, gastrointestinal digestion, anaphylaxis, nsLTP, IgE binding

## Abstract

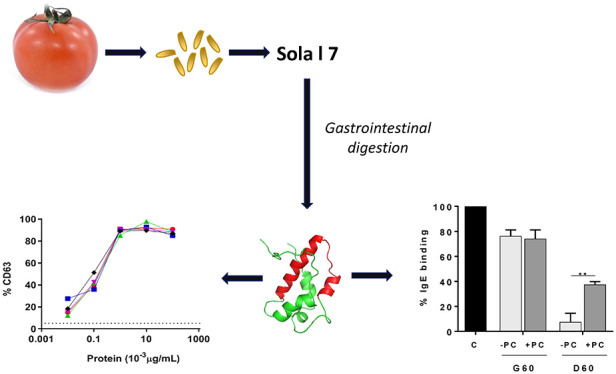

The influence of gastrointestinal digestion on the immunological properties of three different nonspecific lipid-transfer proteins (nsLTPs) described in tomato fruit has been assessed using an *in vitro* system mimicking the stomach and intestine digestion conditions. Tomato peel/pulp nsLTP, Sola l 3, was degraded after digestion, although the immunoglobulin E (IgE) recognition of intact protein and a 10 kDa band were still observed after 30 min of duodenal digestion in the presence of phosphatidylcholine. The tomato seed nsLTP, Sola l 7, showed a higher stability than the other seed allergen, Sola l 6, during digestion. Sola l 7 showed an IgE immunoreactive 6.5 kDa band in immunoblotting analysis, retaining up to 7% of its IgE-binding capacity in inhibition ELISA test after 60 min of duodenal digestion and keeping intact its ability to activate basophils after digestion. These results suggest that the tomato seed allergen Sola l 7 might be considered as an important allergen in the induction of allergic responses to tomato due to its high stability against gastrointestinal digestion.

## Introduction

The prevalence of immunoglobulin E (IgE)-mediated allergies has dramatically increased in both developed and developing countries over the last decades, affecting about 25% of the general population.^1^ Food allergies affect 2% and 8% of adult and child populations, respectively,^2,3^ triggering gastrointestinal symptoms, angioedema, respiratory problems, urticaria, and, in the most severe cases, systemic reactions such as anaphylaxis, severely compromising the life of patients.

Nonspecific lipid-transfer protein (nsLTP) allergy is the most frequent cause of primary food allergy and food-induced anaphylactic reactions in the adult population of Mediterranean countries, being as these proteins are important panallergens involved in cross-reactivity processes.^4^ As members of the prolamin superfamily, nsLTPs are small and compact proteins mainly formed by α-helices separated by short turns and firmly anchored by four disulfide bridges.^5^

Pru p 3, the nsLTP from the peach peel, is considered the main sensitizer in nsLTP allergy among the *Rosaceae* fruit family, although other relevant nsLTPs from different plant-food families are also involved in important allergenic processes. In this regard, the tomato (*Solanum lycopersicum*), belonging to the *Solanaceae* family, has been described as one of the most prevalent plant-derived food sensitizers.^6−8^ In the allergogram of tomato fruit, five allergens from the peel and pulp, Sola l 1–Sola l 5, have been identified and characterized to date,^9−11^ and other proteins such as Sola l Glucanase and Sola l Peroxidase have also been detected, although they need deeper characterization.

Recently, numerous studies have focused on the role of seed allergens in triggering severe and unexpected allergic reactions.^12^ In this context, we have previously shown that tomato seed extract recruited a significant IgE reactivity, and we identified two new tomato seed allergens, Sola l 6 and Sola l 7, responsible for severe symptoms (anaphylaxis and angioedema) in the studied population.^13^ Both proteins differ structurally and immunologically to the already described tomato peel/pulp nsLTP, Sola l 3.^13^ Sola l 3 and Sola l 7 belong to class 1 nsLTPs, to which Pru p 3 also belongs, being identified in a multitude of plant sources, trees, weed pollens, and foods and characterized by a long tunnel-like cavity. In contrast, Sola l 6 belong to class 2 nsLTPs, which are proteins with two adjacent hydrophobic cavities, are scarcely studied, and are neither very abundant nor have a high allergenic potency in the few sources described.^14^

Food allergens are usually resistant to the harsh acidic environment of the stomach and resist digestion by gastrointestinal enzymes. Therefore, they can reach the gut-associated lymphoid tissues with still a high immunologic potency.^15−17^ Moreover, nsLTPs can bind lipids through their hydrophobic cavity, which is a key point in the maintenance of allergenicity during the digestion of certain proteins. The interaction of lipids with proteins in solution has been demonstrated to be important in the affecting rates of proteolysis and in particular has provided a protective effect for some milk proteins.^16,18,19^

On the contrary, allergens can be digested into molecular mass peptide fragments big enough to retain the IgE-binding and T-cell stimulating capacity.^20,21^ Because of the difficulties involved in conducting *in vivo* experiments to evaluate the gastrointestinal stability of the different food allergens, *in vitro* digestion models provide an alternative tool.^22,23^

The aim of this work was to provide an immunological characterization of the digestion products of three tomato nsLTPs from the peel and seeds by immunoassays and basophil activation test (BAT). For this purpose, an *in vitro* simulated gastrointestinal digestion was performed using a two step system, which mimics the successive passage through the stomach and duodenum and includes phosphatidylcholine (PC), a physiological surfactant secreted by the stomach mucosa.

## Materials and Methods

### Recombinant and Natural Tomato Proteins

Natural Sola l 3, Sola l 6, and Sola l 7 were purified from Applause tomatoes and characterized as described previously.^13^ Natural tomato allergens were used for skin prick test.

In order to produce the recombinant form of tomato allergenic nsLTPs, we isolated total RNA and with specific primers designed from the fingerprint analysis; we cloned the specific fragment into the PCR 2.1 vector (TOP10 *E. coli* cells). Subsequently, sequences were cloned into the pPICZαA vector and electroporated into *Pichia pastoris* KM71H electrocompetent cells. After 72 h of methanol induction, recombinant tomato proteins were isolated from extracellular culture and purified throughout a DEAE anion exchanged chromatography and a C-18 reverse phase column anchored to a reverse phase high-performance liquid chromatograph (RP-HPLC), with the same conditions as those described for the natural ones.^13^ Protein concentration was determined by a bicinchoninic acid protein assay kit (Pierce Scientific, Rockford, IL) and spectroscopic analysis. The maintenance of the secondary and tertiary structures of recombinant allergens compared with those of the native counterparts and their thermal stability was verified by means circular dichroism (Figure S1) and nuclear magnetic resonance experiments (data not shown).

### Human Sera

Individual blood samples from 13 allergic patients with a proven allergy to tomato were collected from the Allergy Services of Hospital Universitario Regional of Málaga and the Hospital Universitario Infanta Leonor of Madrid. The diagnosis of IgE-mediated tomato allergy was made on the basis of a well-defined clinical history of tomato allergy and a positive skin prick test (SPT) to tomato together with evidence of specific IgE antibodies (sIgE). The SPT with nsLTP (Pru p 3 enriched: ALK-Abelló, Madrid, Spain) was also performed to assess the LTP sensitization. The patients were classified according to their clinical symptoms after tomato intake: mild reactions, including oral allergic syndrome, moderate reactions, such as urticaria, and severe reactions, including anaphylaxis (Table 1).

**Table 1:** Demographic and Clinical Characteristics of the Patients^a^

patient	age (years)	sex	clinical symptoms	tomato	peach peel (Pru p 3)	tomato pulp	tomato seed	Sola l 6/7	sIgE tomato (kU/L)
1	28	F	OAS	**+**	**+**	**+**	**–**	**–**	5.67
2	32	F	Anaph	**+**	**+**	**+**	**–**	**–**	1.66
3	50	F	Anaph	**+**	**+**	**+**	**–**	**–**	1.41
4	21	M	Anaph	**+**	ND	**+**	ND	ND	ND
5	25	F	Urt	**+**	**+**	**+**	**–**	**–**	0.27
6	37	F	Anaph	**+**	**+**	**+**	**+**	**+**	2.16
7	40	F	Anaph	**+**	**+**	**+**	**+**	**+**	19.8
8	17	F	Urt	**+**	**+**	**+**	**+**	ND	2.28
9	23	F	Urt	**+**	**+**	**–**	**+**	**+**	5.12
10	30	F	OAS	**+**	**+**	**+**	**+**	ND	8.36
11	27	F	OAS	**+**	**+**	**+**	**+**	**+**	0.97
12	23	F	OAS	**+**	**+**	**–**	**+**	**+**	2.5
13	33	F	Anaph	**+**	**+**	**+**	**+**	**+**	ND

a F, female; M, male; Anaph, anaphylaxis; Urt, urticaria; OAS, oral allergic syndrome; **+**, positive; −, negative; ND, not determined.

SPT with tomato extract (ALK-Abello, Madrid, Spain) and purified tomato seed nsLTPs described in this study (Sola l 3, Sola l 6, and Sola l 7) at 5 and 50 μg/mL were conducted according to standard procedures.^24^ Moreover, homemade pulp and seed tomato extracts were also used at 5 and 50 μg/mL for total protein concentration. Histamine dihydrochloride (10 mg/mL) and physiologic saline solution were used as positive and negative controls, respectively. SPT responses were read after 15 min, and a wheal size of 3 mm^2^ greater than the negative control was considered positive.

The total tomato sIgE levels in serum samples were determined by ImmunoCAP-FEIA according to manufacturer’s instructions (Thermo Fisher Scientific, Uppsala, Sweden) (Table 1).

A group of age- and sex-matched individuals with a tolerance to tomato and without food allergic symptoms was included as a negative control.

All human samples were obtained according to the principles of the Declaration of Helsinki and approved by the Ethics Committee of both hospitals. All patients signed the corresponding informed consents. The Ethical Committee of the Complutense University (Madrid, Spain) and the Ethical and Research Committee of Hospital Regional Universitario of Malaga (#02/2012) approved the protocols used for this experimental work and all the methodology related to the use of human sera in this study.

### *In Vitro* Gastric and Duodenal Digestion

*In vitro* gastroduodenal digestions of recombinant Sola l 3, Sola l 6, and Sola l 7, using simulated fluids, were performed as described previously.^25^ Briefly, gastric digestion (GD) was conducted in simulated gastric fluid (35 mM NaCl, pH 2) in the absence or presence of phosphatidylcholine (PC) vesicles at 37 °C, with the addition of porcine pepsin (182 U/mg of protein). Phospholipid vesicles were prepared by dissolving egg l-α-phosphatidylcholine from Larodan (Malmo, Sweden) in 35 mM NaCl pH 2.0 at a concentration of 9.58 mg/mL. Then, the solution was sonicated in ice (raising the power from 10% to 50% in 5 min, and keeping it for 5 min at 60% power), not exceeding a sample temperature of 40 °C. Phospholipid vesicles were filtered through Filtropur 0.45 μm of poly(ether sulfone) from Sarstedt (Nümbrecht, Germany) to remove any possible titanium particles. During gastric digestion, aliquots were withdrawn at 0, 15, 30, and 60 min. Duodenal digestion (DD) was performed on the 60 min gastric digests readjusted to pH 6.5, with the addition of 0.25 M Bis-Tris, pH 6.5, 1 M CaCl_2_, and a 0.25 M bile salts mixture, containing equimolar quantities of sodium glycodeoxycholate and sodium taurocholate. After preheating at 37 °C for 15 min, pancreatic porcine lipase (24.7 U/mg protein) and colipase (1:895, w/w), pancreatic bovine trypsin (34.5 U/mg protein), and α-chymotrypsin (34.5 U/mg protein) were added. The reactions were stopped after 30 and 60 min at 37 °C by adding a solution of Bowman–Birk inhibitor. All enzymes and reagents were purchase from Sigma-Aldrich (St. Louis, MO).

### Reverse Phase High-Performance Liquid Chromatography (RP-HPLC)

Native tomato nsLTPs and their gastroduodenal digests were analyzed in a RP-HPLC SHIMADZU (LC 20AB) system using an Ultrasphere reverse-phase C-18 column. Operating conditions were as follows: solvent A, 1 mL/L TFA in Milli-Q water; solvent B, 1 mL/L TFA in HPLC grade acetonitrile; flow rate, 1.5 mL/min; injection volume, 200 μL (1 mg/mL). A linear gradient of solvent B in A, from 0% to 30% in 60 min, followed by 50% B for 20 min was used.

### Human IgE Binding by Inhibition ELISA

Human IgE binding to Sola l 3, Sola l 7, and their digests was assessed by inhibition ELISA as previously reported.^26^ using pooled sera from five (numbers 1–5, Table 1) and seven (numbers 6–12, Table 1) tomato allergic patients, respectively. Data were obtained by registering visible absorption at 492 nm after the addition of *o*-phenylenediamine dihydrochloride as a substrate for horseradish peroxidase. Undigested proteins were used as controls.

### SDS-PAGE Analyses

Intact proteins and their digests were diluted in tricine sample buffer (Bio-Rad, Richmond, CA), with 5% (v/v) of β-mercaptoethanol (β-ME) and were heated at 95 °C for 5 min. Samples were loaded and analyzed on Precast Criterion XT 16.5% Tris-Tricine gels (Bio-Rad). Separations were carried out at 100 V during 1 h and 45 min, using a Tris-Tricine running buffer (Bio-rad) in the Criterion cell. Finally, gels were stained with Bio-Safe Coomassie G-250 (Bio-Rad).

### IgE Immunoblotting

After SDS-PAGE separation, gels were soaked in transfer buffer (48 mM Tris, 39 mM glycine, 20% methanol, pH 9.2) for 20 min and subjected to semidry transfer in a Trans-Blot SD (Bio-Rad) for 30 min at 18 V. The nitrocellulose membranes were blocked with 3% skim milk powder in phosphate-buffered saline, pH 7.4 containing 0.05% Tween 20 PBS-T. The IgE immune detection of the allergenic fragments after gastrointestinal digestions of Sola l 3 and Sola l 7 were carried out with pools of five (numbers 1–5 from Table 1) and seven (numbers 6–12 from Table 1) tomato allergic patient sera (diluted 1:5), respectively, as previously described (Sirvent et al. in 2014).^12^ Briefly, the binding of human IgE was detected by using the mouse anti-human IgE antibodies (diluted 1:5000) kindly donated by Alk-Abelló, followed by horseradish peroxidase (HRP)-labeled rabbit anti-mouse IgG (diluted 1:5000) (DAKO, Glostrup, Denmark). The signal was developed with a chemiluminescent ECL-Western blotting reagent (GE Healthcare, Chicago, IL).

### Basophil Activation Test

The basophil activation test was performed using 100 mL of heparinized whole blood, incubated with 20 μL of stimulation buffer (1 M HEPES buffer containing 0.78% NaCl (w/v), 0.037% KCl (w/v);, 0.078% CaCl_2_ (w/v), 0.033% MgCl_2_ (w/v), 0.1% HSA (w/v), 10 μL/mL of IL-3 (1 mg/mL), and 1 μL of monoclonal antibody CCR3-APC (1 mg/mL) (BioLegend INC, San Diego, CA) for 10 min at 37 °C. Then, 100 μL of each purified Sola l 7, Sola l 6, and their digests was added at different concentrations following a 10-fold dilution pattern (0.1–0.00001 μg/mL) and incubated for 30 min at 37 °C. Anti-human IgE (0.5 mg/mL) (BD Biosciences, Franklin Lakes, NY) or PBS were used as a positive and negative controls, respectively. After 5 min on ice to stop the degranulation process, samples were incubated with 1 mg/mL of monoclonal antibodies anti-CD 203c-PE and CD63 FITC (BioLegend) for 15–20 min at 4 °C. Finally, lysis solution (BD Biosciences) was used for red cells disruption. Cells were analyzed using a FACSCalibur flow cytometer (BD Biosciences), acquiring at least 500 basophils per sample. Results are presented as the percentage of activated basophils (CD63^+^CD203c^+^CCR3^+^). Due to the low availability of patients, only the sera of one tomato allergic patient sensitized to Sola l 6 and Sola l 7 and displaying anaphylaxis (patient numbers 13 and 7, respectively) was used for this experiment.

## Results and Discussion

### Recombinant Protein Production

In order to demonstrate the similarity of the recombinant proteins with their natural analogues, the content in the secondary structure of purified recombinant proteins was spectroscopically determined by circular dichroism (CD) (Figure S1). The three proteins presented the typical characteristics of α-helix-enriched proteins, as nsLTPs are, with a secondary structure showing two clearly marked minima at 208 and 220 nm.

Allergenic nsLTPs are stable until 80 °C, and they recover almost completely their secondary structure after heating. rSola l 3 presented the most stable secondary structure of all the allergenic tomato nsLTPs analyzed. rSola l 6 was the nsLTP with the lowest molar ellipticity per residue, and in the case of rSola l 7, the seed class 1 nsLTP recovered the structure after being restored to the original conditions.

Sola l 7 is produced in the seeds as a trimeric form, showing the same IgE recognition by sensitized patients as the monomeric one when was treated with a reducing agent, such as β-ME (Figure S2). Interestingly, the recombinant form was produced in the same trimeric form by the Pichia yeast, showing the same degree of recognition by a pool of sera from allergic patients.

### *In Vitro* Simulated Gastrointestinal Digestion

**Figure 1:**
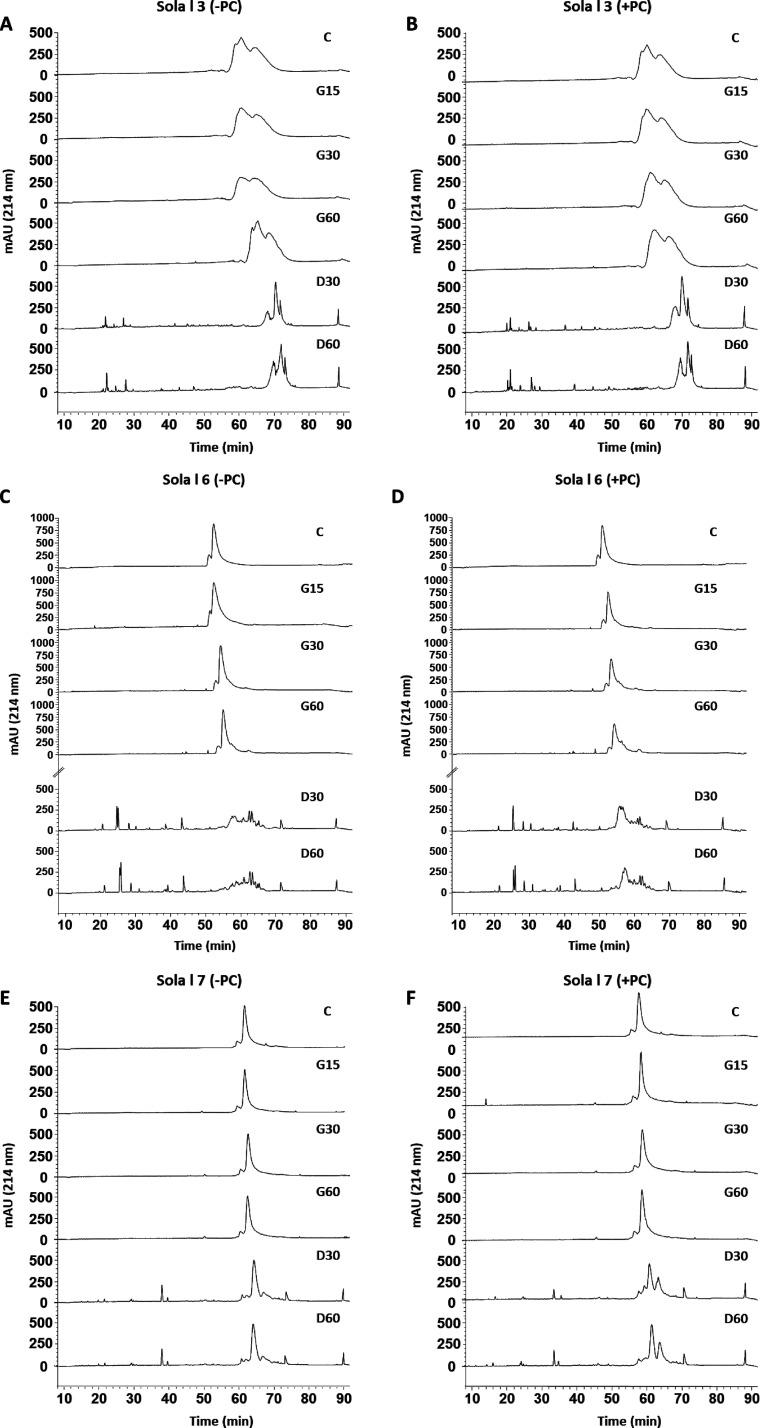
RP-HPLC analysis of (A and B) Sola l 3, (C and D) Sola l 6, (E and F) Sola l 7, and their digestion products. Digestions were performed in the (A, C, and E) absence or (B, D, and F) presence of PC. C, undigested protein control; G15, 15 min gastric digests; G30, 30 min gastric digests; G60, 60 min gastric digests; D30, 60 min of gastric followed by 30 min of duodenal digestion; D60, 60 min of gastric followed by 60 min of duodenal digestion.

The RP-HPLC analyses of the three tomato nsLTPs following the *in vitro* gastric (GD) and duodenal (DD) digestions are shown in Figure 1. To assess the effect of the interaction between proteins and lipids, *in vitro* GD was performed in the absence and presence of PC, a physiological lipid component secreted by the gastric mucosa and also present in the bile during digestion. On the basis of proteomic results, tomato peel nsLTP, Sola l 3, did not show significant changes compared to undigested protein during GD (Figure 1A), although its digestibility is slightly increased in the duodenal phase, with no significant changes with the presence of PC (Figure 1B).

Sola l 7 is the tomato seed allergenic protein that showed greater stability after digestion.

**Figure 2:**
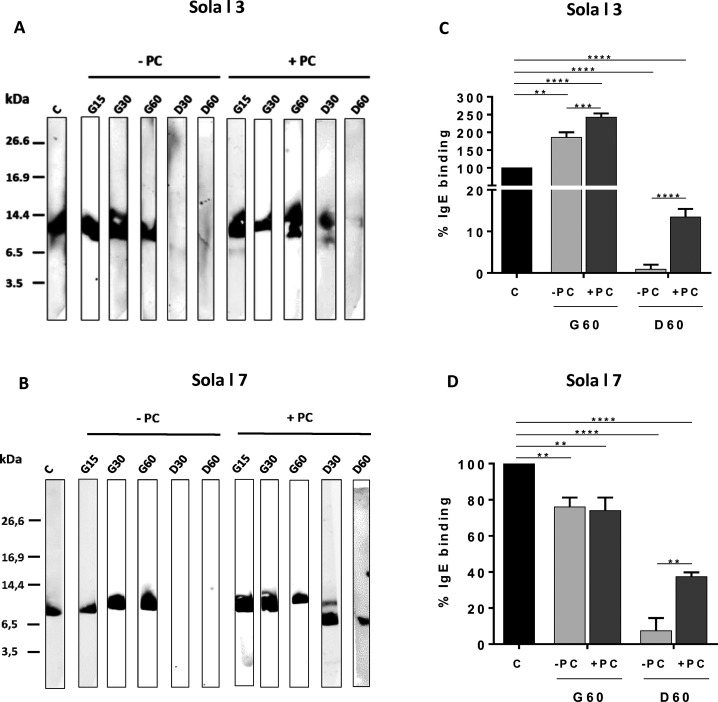
(A and C) Western blotting and (B and D) IgE inhibition ELISA of (A and B) Sola l 3 and (C and D) Sola l 7 after *in vitro* gastroduodenal digestions in the presence (+ PC) or absence (− PC) of PC. Molecular mass marker containing triosephosphate isomerase (26.6 kDa), myoglobin (16.9 kDa), α-lactalbumin (14.4 kDa), aprotinin (6.5), insulin b chain (3.4 kDa), and bacitracin (1.4 kDa). C, control undigested protein; G15, G30, and G60, 15, 30 and 60 min gastric digests, respectively; D30 and D60, 60 min of gastric followed by 30 and 60 min of duodenal digestion, respectively. Pool sera from patients (A and B) 1–5 and (C and D) 6–12, described in Table 1, were used. **<0.01, ***<0.001, ****<0.0001. Error bars represent SEM of three independent experiments.

A stable chromatographic peak was observed through the GD (Figure 1E), corresponding to a band of 10 kDa, as was observed by SDS-PAGE electrophoresis (data not shown) and immunoblotting (Figure 2C), which was degraded into a 6.5 kDa fragment when simulated duodenal fluid in the presence of PC was performed (Figure 1F).

The comparison of the chromatographic profile of the tomato seed nsLTP, Sola l 6, after *in vitro* gastrointestinal digestion in the absence (Figure 1C) and presence (Figure 1D) of PC showed that the presence of lipids decreased the protein resistance to hydrolysis with pepsin. However, PC slightly increased the protein protection to hydrolysis during DD, detecting a lower content of degradation products.

These results are consistent with the presence of intact protein at the end of gastric digestion of the three studied proteins, as it was seldom cleaved by pepsin, and their degradation occurs at the end of the duodenal stage. A similar high stability to pepsinolysis during simulated gastric digestion has been reported for other nsLTPs such as peach,^27^ cherry,^28^ grape,^29^ or sunflower.^30^ The ability of a nsLTP from sunflower to bind PC protects the protein against digestive enzymes,^30^ slowing down proteolysis. Proteins from other families have also demonstrated an increased stability against digestion in the presence of PC such as lysozyme,^26^ α-lactalbumin,^18^ or β-lactoglobulin,^31^ which interact with the surfactant *via* the secondary fatty acid binding site in the hydrophobic groove along the single strand of its α-helix.^32^ The duodenal digestion of nsLTPs has been studied to a lesser extent, although results for the nsLTPs from peach^27^ and sunflower^30^ indicate that they also show a high resistance to duodenal enzymes, which was enhanced by the addition of PC.

In contrast, the gastric and duodenal digestion of grape nsLTP was not influenced by the presence of the lipid ligand PC,^29^ and an interaction with lipids slightly increased the susceptibility of wheat nsLTP to gastroduodenal digestion because of changes in its tertiary structure.^33^

The stable three-dimensional (3D) conformation of class 1 nsLTPs, and the presence of an extensive disulfide bond core, makes these allergens exceptionally resistant to thermal or enzymatic degradation, and it is thought to be one of the most important factors to maintain the 3D structure of a protein contributing to the severe systemic reactions often observed in allergic patients.^34^ The presence of disulfide bonds also affects the resistance of allergens to digestion, such as in the case of grape nsLTP or a Pru p 3-like folding variant.^29,35^ Class 2 nsLTPs are well-known for their less stable structure, making them more susceptible to thermal and enzymatic processing.^36^ In accordance with this fact, *in vitro* gastrointestinal digestion conducted with Sola l 6 showed a higher hydrolysis than that produced with Sola l 3 and Sola l 7.

### IgE Immunoreactivity of Tomato nsLTPs after *in Vitro* Digestion

In order to compare the IgE reactivity of digestion products, samples were evaluated by direct immunoblotting using a pool of sera from tomato allergic patients. Due to the limited amount of serum available for the other tomato nsLTPs, we selected digested samples from Sola l 7 among seed nsLTPs based on its higher resistant to digestion. The immunoblotting of gastric and duodenal digests in the absence and presence of PC is shown in Figure 2.

The IgE binding of Sola l 3 fell drastically at the duodenal phase in the absence of PC. However, after 30 min of DD in the presence of PC, the IgE recognition of intact protein is still observed as well as a 10 kDa band (Figure 2A). Intact Sola l 7 exhibited a considerable IgE-binding activity, which virtually disappeared after DD in the absence of PC. In the presence of PC, IgE binding to intact protein was detected even after 30 min of DD. A band of 6.5 kDa remains immunoreactive after 60 min of DD (Figure 2B).

We further checked the samples by inhibition ELISA with the same pool of sera as in the immunoblotting. The immunoreactivity of Sola l 3 increased at the end of the GD, showing values of 185% of IgE binding in the absence of PC and even higher in the presence of PC, reaching values of 241% compared to the undigested protein and indicating that the unfolding of the protein during the gastric phase would expose IgE epitopes (Figure 2C). It is expected that conformational epitopes are more susceptible to be damaged than sequential epitopes,^37^ and it has been described that the partial unfolding under the acidic gastric conditions, could enhance an effective allergic response, likely because it unmasks B-cell epitopes.^38^ In agreement with immunoblotting data, IgE reactivity fell drastically at the end of DD until values close to zero when the digestion was performed in the absence of PC. When PC was included in the simulated digestion, the IgE reactivity remained close to 13%, compared to the undigested protein, showing the protective effect of PC on the enzymatic degradation of Sola l 3.

In contrast, Sola l 7 retained 7% of IgE-binding capacity after gastroduodenal digestion in the absence of PC (Figure 2D) despite no signal was detected by immunoblotting, probably due to differences in the physicochemical properties of allergen molecules in liquid (ELISA) versus solid (immunoblotting) phases. Similar results have been described for other proteins where the loss of the tertiary structure, with the subsequent loss of conformational epitopes by the disruption of intramolecular disulfide bonds could decrease or even abolish their allergenicity.^39^ Examples are the easily digestible pollen-related food allergens, such as Mal d 1 (apple), Api f 1 (celery), and Cor a 1 (hazelnut) belonging to Bet v 1-like family, which completely lose their ability to bind IgE in the first minutes of GD/DD. As it has been shown with Sola l 3, the presence of PC during digestion exerted a protective effect against the degradation of Sola l 7, preserving immunoreactivity values against IgE at the end of DD of up to 37% compared to that of undigested protein.

The structural compactness of nsLTPs is attributed to the presence of a conserved skeleton of cysteine residues that forms multiple disulfide bonds. However, nsLTPs allergens also present type 1 (linear) epitopes, according to ample literature consensus. This composition gives these allergens excellent properties to resist digestion and heat treatment,^40^ preserving almost the whole core of the molecule as an immunogenic fragment, as observed at the end of the DD of Sola l 7 (Figure S3).

### *Ex Vivo* Biological Activity

**Figure 3:**
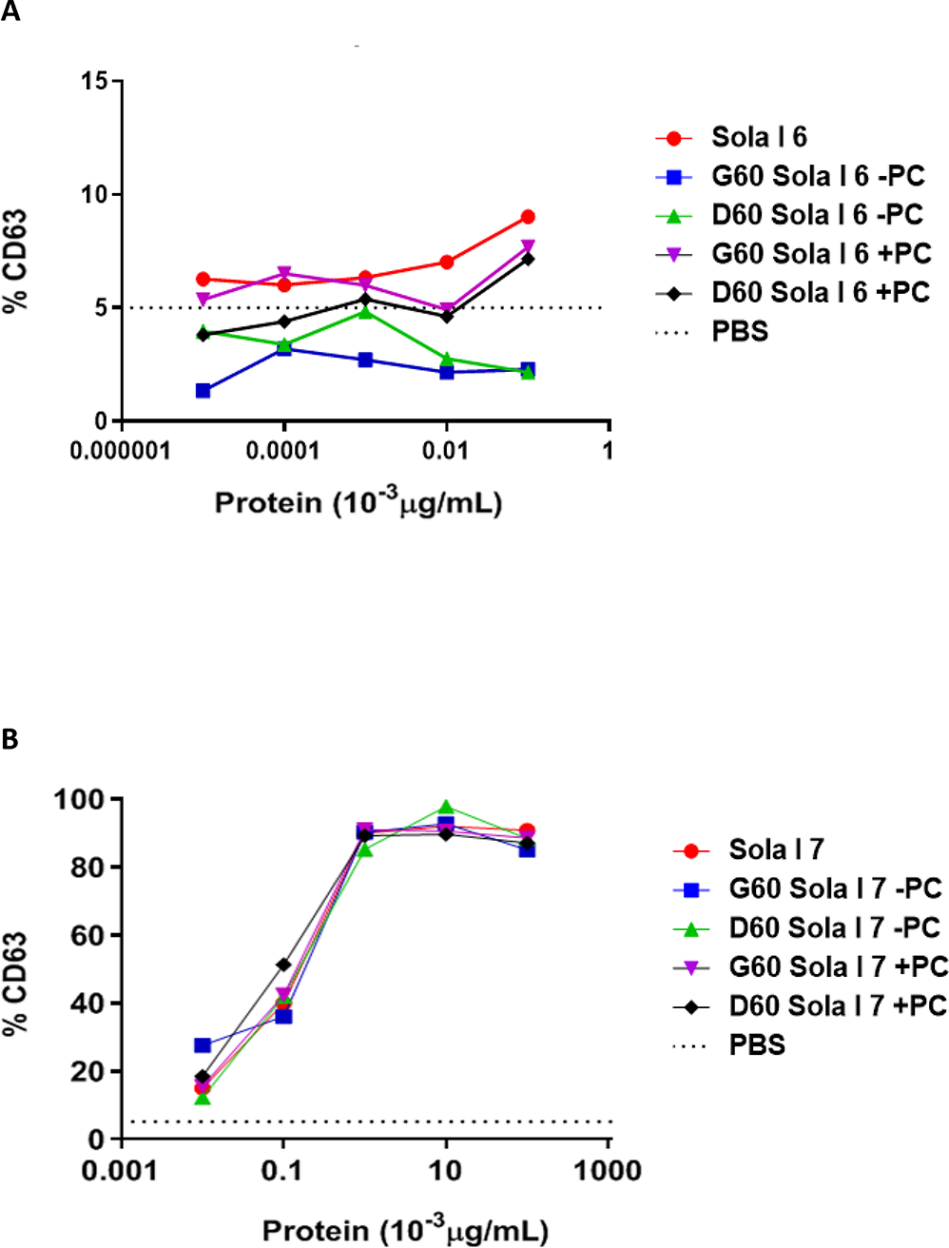
Percentage of activated basophils (CD63^+^CD203c^+^CCR3^+^) after stimulation with (A) Sola l 6 and (B) Sola l 7 and its 60 min gastric and 60 min duodenal digests obtained in the presence and absence of PC. Whole blood from one tomato allergic patient sensitized to Sola l 6 and Sola l 7 was used (numbers 13 and 7, respectively, Table 1).

To study, in-depth, the repercussion of the digestion on the allergenicity of tomato seed allergens, Sola l 6 and Sola l 7, and in order to corroborate the data obtained from the immunoblotting and the ELISA for Sola l 7 digests, the allergenic capacity of the intact proteins and their digests obtained in the presence and absence of PC was evaluated *ex vivo* by performing a basophil activation test (Figure 3). The ability of intact Sola l 6 to activate basophils was much weaker than that of Sola l 7. The digests of Sola l 6 obtained in the absence of PC impaired the activation of basophils, while the digested protein obtained in the presence of PC induced the activation of basophils, again indicating the protective role of PC in the enzymatic degradation of tomato LTPs. In contrast, the gastrointestinal digestion of Sola l 7 does not affect its ability to activate effector cells, being the percentage of activated basophils similar to that of the undigested control protein regardless of the presence of PC during digestion, and despite the fact that no IgE recognition was observed by immunoblotting. These data indicates that Sola l 7 is an important allergen to be considered during allergic sensitization, showing a high immunogenicity and resisting the gastrointestinal digestion. In addition, it should be noted that, due to the lack of patient availability, only the serum of one tomato allergic patient sensitized to Sola l 6 and Sola l 7 was used for this experiment. Discrepancies between the *in vitro* digestibility and allergenicity of certain allergens has been reported,^41^ maybe because the *in vitro* digestibility of a protein is influenced by the hydrolysis conditions and enzyme quantities, not considering the protein interactions with other digestive or food matrix components. In addition, even though certain proteins are consistently degraded in the *in vitro* assays, it cannot be discarded that, in an *in vivo* situation, minimal amounts of intact material in an immunologically active form bypass the digestion.^42^ In our case, the discrepancies found between the *in vitro* studies performed on Sola l 7, with a maximum of 7% IgE binding, and in *ex vivo* experiments may be due to the fact that the second technique is more sensitive and the degradation products are in liquid phase, being exposed all the IgE epitopes of the protein, either conformational or linear ones. For this reason, a complete study at different levels is essential for this type of approach.

In summary, we provided for the first time the *in vitro* gastrointestinal digestion of three nsLTPs described in tomato fruit with a high allergenic potential. Tomato peel nsLTP, Sola l 3, showed partial resistance to gastrointestinal digestion (Figure 1A,B), which involved an immunogenic response in the duodenal phase in the presence of PC. Similarly, class 2 tomato nsLTP, Sola l 6, was also completely hydrolyzed after gastric digestion, although the presence of PC conferred resistance to the degradation in the early stages of duodenal digestion. Sola l 7 allergen is described as the tomato nsLTP most resistant to gastric and duodenal digestion in the presence of PC, generating an allergenic 6.5 kDa fragment that can trigger an immune response.

In this manuscript, we suggest that the tomato seed allergen Sola l 7 might be an important allergen to be considered during allergic sensitization and/or allergic responses to tomato due to its high immunogenicity and stability against gastrointestinal digestion. To date, only allergens from the tomato fruit have been reported. The identification and characterization of two new seed allergens could facilitate the tomato allergy diagnosis. Usually, only the pulp is applied to carry out the skin prick test used as a diagnostic test, and therefore, some patients reactive to the seed proteins could be considered as false negatives. The inclusion of Sola l 7 in diagnostic tests could improve the diagnosis of tomato allergy since seeds are included in many processed foods, and tomato seed allergens, as Sola l 7, are described as responsible for anaphylactic shock during ingestion.^13^ The in-depth characterization carried out in this work will allow for advancement in the knowledge of tomato seed allergens, of which their use should be considered in clinical diagnosis and to establish consumption patterns for tomato allergic patients.

## Supporting Information Available

Figure of circular dichroism spectra of recombinant Sola l 3, Sola l 6, and Sola l 7 (PDF)
Figure of SDS-PAGE patterns of natural and recombinant Sola l 7 (PDF)
Figure of 3D modeling of the 6.5 kDa immunogenic fragment (PDF)

## Abbreviations Used

nsLTP: nonspecific lipid-transfer protein
SPT: skin prick test
DD: duodenal digestion
GD: gastric digestion
PC: phosphatidylcholine
sIgE: specific immunoglobulin E
