# Supplementary figures and images for: IgE-Reactivity Pattern of Tomato Seed and Peel Nonspecific Lipid-Transfer Proteins after in Vitro Gastrointestinal Digestion
Source: J Agric Food Chem. 2021 Mar 15;69(11):3511–8. doi: 10.1021/acs.jafc.0c06949 (PMC9134490; doi:10.1021/acs.jafc.0c06949)

Supplementary Figure 1

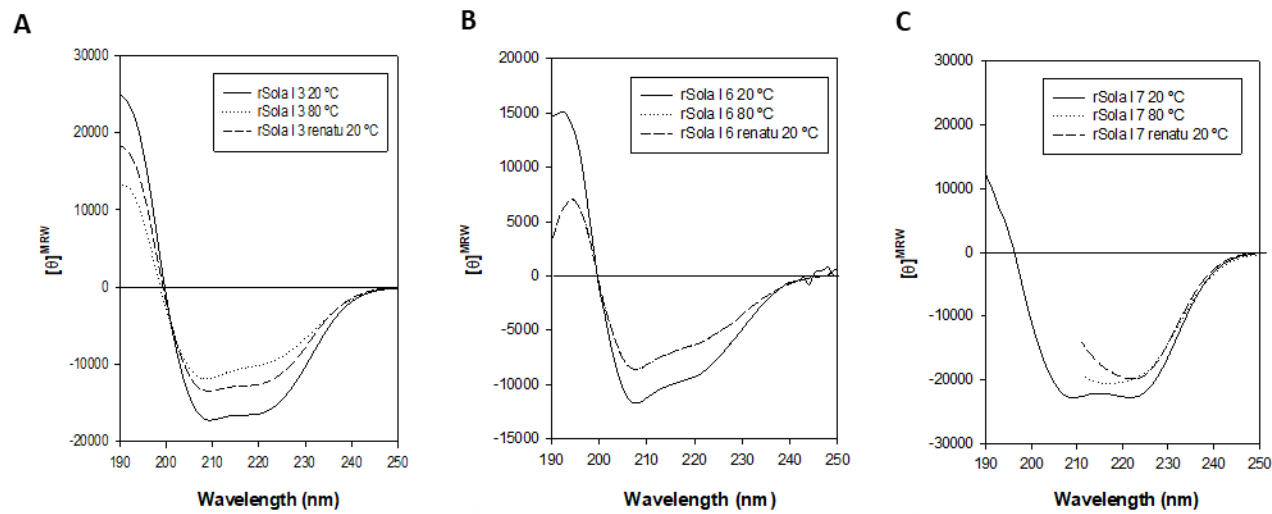

Supplement: Supplementary file 1 — jf0c06949_si_001.pdf [file jf0c06949_si_001.pdf]

Supplementary Figure 2

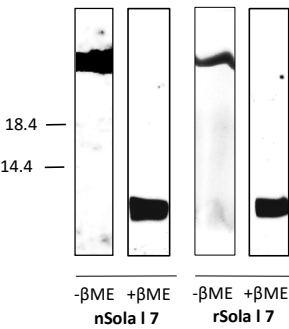

Supplement: Supplementary file 2 — jf0c06949_si_002.pdf [file jf0c06949_si_002.pdf]

**Supplementary Figure 3**

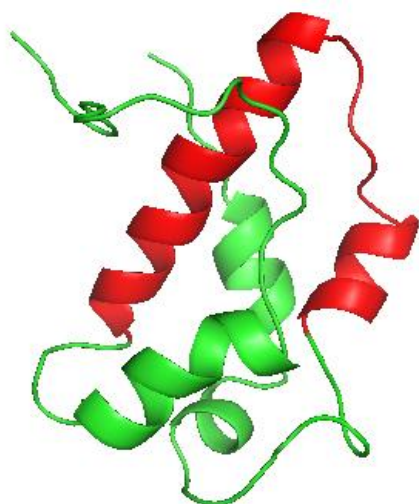

Supplement: Supplementary file 3 — jf0c06949_si_003.pdf [file jf0c06949_si_003.pdf]
